# Association between triglyceride-glucose index and papillary thyroid carcinoma among Chinese adults with thyroid nodules

**DOI:** 10.3389/fendo.2025.1616350

**Published:** 2025-09-25

**Authors:** Chunyan Song, Miaomiao Ping, Ling Lin, Xing Meng, Yun Lan, HuaCheng Tong

**Affiliations:** Clinical Laboratory, Nanjing Tongren Hospital, School of Medicine, Southeast University, Nanjing, China

**Keywords:** triglyceride, glucose, insulin resistance, papillary thyroid carcinoma, Chinese adults

## Abstract

**Background:**

Growing evidence has demonstrated that the Triglyceride-Glucose (TyG) index, a reliable and widely recognized marker of insulin resistance, is strongly associated with the development various of types of cancer. For instance, previous studies have demonstrated that elevated TyG index levels are significantly associated to an increased risk of different cancers. Insulin resistance, as reflected by the TyG index, may contribute to tumorigenesis through multiple pathways, including promoting cell proliferation, angiogenesis, and inhibiting apoptosis. Despite these findings, research on the association between the TyG index and papillary thyroid carcinoma(PTC) in Chinese populations is scarce. Given the rising thyroid malignancy incidence, clarifying this relationship is crucial for clinical and public health.

**Objective:**

To explore the association between the TyG index and papillary thyroid carcinoma prevalence.

**Methods:**

This cross-sectional study included patients who underwent fine-needle aspiration of thyroid nodules at Nanjing Tongren Hospital from June 2018 to December 2024. A multivariate logistic regression model was used to examine the association between the TyG index and papillary thyroid carcinoma. Furthermore, stratification and interaction analyses were performed to assess the stability of the association across various subgroups. Moreover, sensitivity analysis further confirmed the stability of the findings.

**Results:**

This study ultimately enrolled 396 patients (mean age 47.8 ± 12.7 years,71.0% females), with the TyG index odds ratio increasing across tertiles. Compared to T1, adjusted ORs for T2 and T3 in papillary thyroid carcinoma were 1.28 and 3.37, respectively. Subgroup and sensitivity analyses supported the results.

**Conclusions:**

This study suggests that TyG index may serve as a valid biomarker for the prediction of papillary thyroid carcinoma in patients with thyroid nodules, although large prospective studies are needed to confirm these findings.

## Introduction

Papillary thyroid carcinoma (PTC) represents the most prevalent form of thyroid malignancy globally, comprising over 80% of all thyroid cancer cases (1). Globally, thyroid cancer ranked 9th among all malignancies in 2020, with an estimated 586,000 new cases (age-standardized incidence 10.1/100,000 in women and 3.1/100,000 in men) (2).Its global incidence has risen 20-30% over the past decade, largely driven by diagnostic advancements and screening uptake, with disproportionate increases among women (2–4). Notably, epidemiological data from China also indicate a significant rise in PTC incidence over the past few decades (5–7). In China, the 2015 National Cancer Registry reported an incidence of 14.6/100,000, ranking 7th among all malignant tumors; among Chinese women, the rate reached 22.56/100,000, placing thyroid cancer 4^th^ (8). Between 1990 and 2019, the age-standardized incidence of thyroid cancer among Chinese women rose from 1.52 to 2.41/100,000, an increase of 58.6%(AAPC + 1.7%, P < 0.001) (9). While PTC generally carries a favorable prognosis, its underlying pathogenesis remains incompletely understood. In particular, the potential role of metabolic factors in the initiation and progression of PTC has recently gained increasing attention (10, 11).

The TyG index has emerged as a novel metabolic biomarker, gaining traction in various disease, including malignancies (12–14). It is a robust biomarker of metabolic health (15). It is an integrated measure of the insulin–glucose–lipid axis that simultaneously captures peripheral insulin resistance and the dynamic imbalance between hepatic VLDL output and β-cell compensation. Derived from fasting serum triglyceride and blood glucose levels, this composite metric reflects insulin resistance and systemic metabolic dysregulation, thus critically assessing overall physiological homeostasis and metabolic function (15). Recent studies have robustly validated the predictive utility of the TyG index across a spectrum of metabolic disorders, including cardiovascular diseases, type 2 diabetes mellitus, non-alcoholic hepatic steatosis and arterial stiffness (baPWV) (16–20). The TyG index is independently associated with arterial stiffness, a key predictor of both cardiometabolic diseases and carcinogenesis. This finding suggests that the TyG index may serve as a surrogate indicator of subclinical vascular damage, thereby linking metabolic dysfunction to long-term cardiometabolic and oncologic outcomes.

Due to its simplicity and reliability, the TyG index has been used as a metabolic indicator to predict disease risk. In the field of oncology, a previous study has used TyG index classification based on tertiles to evaluate its relationship with endometrial carcinoma risk (21). The study found that the risk of endometrial cancer was significantly higher in patients in the highest tertile group. Beyond malignancy, emerging research has also revealed a potential association between the TyG index and Post-COVID-19 syndrome (PCS). Fierro et al.’s retrospective cohort study confirmed a significant association between PCS and insulin resistance(assessed by TyG index) (22). Additionally, growing evidence highlights that the TyG index is not only strongly correlated with metabolic syndrome (23)but may also contribute to the pathogenesis and progression of multiple malignancies (12–14). However, the relationship between the TyG index and metabolic syndrome has yielded heterogeneous findings. While several investigations have reported a strong positive association, other studies have observed a modest or even non-significant correlation after adjusting for confounders such as BMI and age (24). These discrepancies underscore the need for population-specific validation and careful interpretation of TyG index thresholds.

While the TyG index has been extensively investigated across a range of diseases, its potential role in thyroid cancer, particularly PTC, remains underexplored. Previous studies have demonstrated elevated TyG index levels in patients with PTC (25); however, data specific to the Chinese population remain limited. Given the escalating incidence of thyroid cancer and the high prevalence of metabolic syndrome in China (6, 26), elucidating the association between the TyG index and PTC is of paramount importance. This study aimed to evaluate whether TyG index may represent a valid biomarker for the prediction of PTC in a Chinese adult population with thyroid nodules. To address this research gap, we conducted a retrospective cross-sectional study involving a cohort of 396 Chinese adults. Since our study employed a cross-sectional design, it is crucial to emphasize that no causal relationships can be inferred. When interpreting the results, this inherent limitation should be carefully considered. Further prospective studies are needed to elucidate causal associations and comprehensively understand the clinical significance of the TyG index in various diseases.

## Materials and methods

### Study design and population

A retrospective analysis was conducted on 396 patients with thyroid nodules who underwent thyroid fine-needle aspiration cytology (FNAC) at Nanjing Tongren Hospital from June 2018 to December 2024. FNAC for thyroid nodules was performed by physicians with specialized puncture training and clinical experience. The patient was placed supine with shoulders elevated to expose the neck, which was disinfected three times with povidone-iodine (each disinfection pass expanded the scope gradually to ensure a sterile field) and was draped with a sterile towel. Anesthesia was generally unnecessary, but 2% lidocaine infiltration was used if needed. A 25G needle attached to a 10 mL syringe was guided by high-frequency ultrasound (linear probe, 7–12 MHz) to insert into the nodule parenchyma, avoiding blood vessels. The plunger was retracted to 5–8 mL for negative pressure, and the needle was moved back and forth 3–5 times with small amplitude (0.5-1.0 cm) before releasing pressure and withdrawing. If sample volume was low, the procedure was repeated 2–3 times. The needle contents were expelled onto 2–3 slides, were smeared at 45° with another slide, and were immediately fixed with 95% ethanol wet fixation for 10–15 minutes. Pathologists evaluated cellular morphology and nuclear atypia in stained smears to determine lesion nature via FNAC. Exclusion criteria were the following:

- individuals with missing data on the TyG index(composed of fasting triglyceride and blood glucose levels);
- patients with a history of thyroid surgery or other tumors;
- patients diagnosed with other malignancies;
- patients using statins and other lipid-lowering medications;
- individuals with diabetes mellitus.

**Figure 1:**
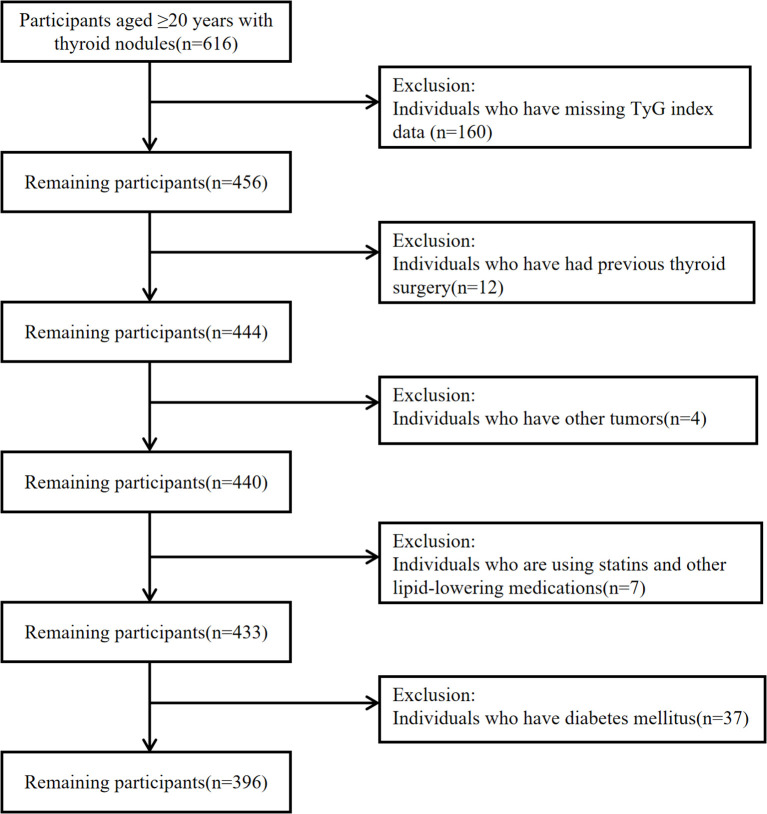
Flow chart of the study.

Participant selection and exclusion criteria are illustrated in **Figure 1**. This study was conducted in accordance with the STROBE guidelines.

To address missing covariate data, a multivariate single imputation approach was employed to derive an unbiased estimate of the association between the TyG index and PTC. Specifically, an iterative imputation method was utilized, with a Bayesian Ridge model serving as the estimator at each step of the round-robin imputation process (27). For comparative validation, all analyses were repeated using the complete data cohort. Furthermore, a series of sensitivity analyses were conducted to evaluate the robustness of the study findings. Sensitivity analysis (SA) is defined as a methodological approach to assess the stability of results by examining the extent to which they are influenced by variations in methods, models, unmeasured variables, or assumptions. This process aims to identify outcomes that are most reliant on potentially questionable or unsupported assumptions (28).

Ultimately, a total of 396 participants were incorporated into the analysis. This research was permitted by the Research Ethics Board of Nanjing Tongren Hospital in February 2025 (Ethics number: 2025-03-020-K001). There was no need for obtaining informed consent since this study retrospectively analyzed existing management and clinical data.

### Data collection

Demographic and clinical parameters of the study participants, encompassing age, sex, vital signs(heart rate, respiratory rate, systolic and diastolic blood pressure), BMI, nodule characteristics(nodule aspect ratio and nodule size), and comorbidities were systematically retrieved from the hospital’s electronic medical record (EMR) system. The nodule aspect ratio and nodule size were obtained from the ultrasound department. Nodule aspect ratio is defined as the ratio of the anteroposterior diameter (longitudinal diameter) to the transverse diameter (horizontal diameter) on ultrasound images. When nodule aspect ratio ≥1 (29), it suggests that the nodule may exhibit a “vertical growth” pattern, which warrants vigilance for malignant potential. Nodule size was determined by measuring the maximum diameter of each thyroid nodule in the longitudinal, transverse, and anteroposterior directions on ultrasound images. A threshold of 1 cm was used as the cut-off value for classifying nodule size (30). The comorbidities include “other nodules”, which refer to any nodules detected outside the thyroid gland in patients with thyroid nodules, such as pulmonary nodules. Concurrently, biochemical parameters, including intact parathyroid hormone (iPTH), thyroid function markers (free triiodothyronine [FT3], free thyroxine [FT4], and thyroid-stimulating hormone [TSH]), serum calcium (Ca), liver enzymes (alanine aminotransferase [ALT], alkaline phosphatase [ALP], and gamma-glutamyl transferase[GGT]), and metabolic parameters(uric acid [UA], total cholesterol [CHOL], triglycerides [TG], and fasting blood glucose [GLU]), were obtained from the laboratory information system (LIS). All laboratory analysis data of patients with thyroid nodules were analyzed using the baseline values of fasting samples collected within 24 hours after admission.

### Laboratory analysis

The TyG index was calculated using the formula: ln[(fasting blood glucose (mmol/L)×18)×(fasting serum triglyceride (mmol/L)×88.5)/2], as previously described (31). Measurements of thyroid function markers, including free triiodothyronine (FT3), free thyroxine (FT4), thyroid-stimulating hormone (TSH), and intact parathyroid hormone(iPTH), were performed using the Roche E602 electrochemiluminescence(ECL) analyzer. Additionally, biochemical parameters, such as serum calcium(Ca), alanine aminotransferase(ALT), alkaline phosphatase(ALP), gamma-glutamyl transferase(GGT), uric acid(UA), total cholesterol(CHOL), triglycerides(TG), and fasting blood glucose(GLU), were quantified using the Roche C701 biochemical analyzer. All assays were conducted using Roche-matched reagents to ensure consistency and reliability. Rigorous daily quality control measures were implemented for both instruments to maintain the accuracy and precision of the results. All procedures adhered strictly to the manufacturer’s standard operating protocols to ensure methodological consistency and reproducibility.

### Statistical analysis

The objective of this study is to investigate the relationship between the TyG index and patients with PTC. Participants were categorized into three groups based on TyG index tertiles. Descriptive statistical analysis was conducted for all enrolled subjects. Normally distributed continuous variables are presented as mean ± standard deviation (SD), while non-normally distributed continuous variables are described using median values with interquartile ranges (IQR). Categorical variables are expressed as frequencies with corresponding percentages (%). To evaluate differences across various groups, we utilized the chi-square test for categorical variables, the one-way analysis of variance (one-way ANOVA; in case of normal distribution), and the Kruskal-Wallis H test (in case of non-normal distribution).

We employed both univariate and multivariate binary logistic regression analyses to investigate the association between variable TyG index and outcome PTC. Model 1 was adjusted for age, sex, heart rate, respiratory rate, BMI. Model 2 was further adjusted for nodule aspect ratio and nodule size. Model 3 was further adjusted for hypertension, other nodules and Hashimoto’s thyroiditis. Model 4 was fully adjusted for iPTH, FT3, FT4, TSH, ALT, ALP, UA, CHOL. Collinearity was evaluated before the multivariate analysis. Covariates were selected based on clinical significance, statistical significance in univariate analysis(p<0.1), and an estimated variable change of at least 10% for potential confounding effects. Age and gender were regularly adjusted. A restricted cubic spline model (a fitted smooth curve) was used to determine the dose-response relationship between the TyG index and PTC. Additionally, potential modifications in the association between the TyG index and PTC were evaluated, including the following variables: age (<45years vs. ≥45years), sex, TSH(<2.778μIU/mL vs. ≥2.778μIU/mL), BMI(<24kg/m² vs. ≥24kg/m²), and hypertension(Yes vs. No). Heterogeneity across subgroups was assessed via multivariate logistic regression, and interactions between subgroups and the TyG index were examined using likelihood ratio tests.

Statistical analysis was performed using R statistical software-version 4.2.2 (http://www.Rproject.org; The R Foundation, Vienna, Austria) and the Free Statistics software-version 2.1.1 (https://www.clinicalscientists.cn/freestatistics/; Beijing FreeClinical Medical Technology Co., Ltd, Beijing, China). A two-sided p-value of less than 0.05 was considered statistically significant.

## Results

### Baseline characteristics of the study participants by categories of TyG index

The TyG index tertile cut-offs were based on our study population’s distribution, consistent with prior research (21). This method allows for a more intuitive assessment of the relationship between the TyG index and PTC risk across different levels of insulin resistance and metabolic disorders. In this study, the TyG index was divided into three tertiles: T1(≤8.184), T2(8.186-8.669), and T3(≥8.679). A total of 396 eligible participants were included, with a mean age of 47.8 ± 12.7 years. Among these participants, the overall prevalence of PTC was 54.8%. **Table 1** illustrates the baseline characteristics of the study population stratified by TyG index tertiles. Our analysis revealed several key differences among the groups. Age showed a progressive increase from T1 to T3 (44.3 ± 12.6 vs. 50.8 ± 11.9 years, *p*<0.001), indicating that higher TyG index levels were associated with older age, a known risk factor for metabolic dysfunction. The proportion of male participants increased significantly with TyG index tertiles(29.0% in T1 vs. 41.7% in T3, *p*<0.001), suggesting potential gender-specific differences in the association between insulin resistance and metabolic parameters. Systolic blood pressure (SBP) and diastolic blood pressure (DBP) increased progressively from T1 to T3(122.3 ± 14.0 vs. 134.2 ± 17.6mmHg for SBP, *p*<0.001; 77.1 ± 10.5 vs. 84.3 ± 11.1mmHg for DBP, *p*<0.001), with hypertension prevalence (27.3%) rising in parallel with TyG index levels. Furthermore, several biochemical parameters, including serum calcium(Ca), alanine aminotransferase(ALT), alkaline phosphatase(ALP), gamma-glutamyl transferase(GGT), uric acid(UA), and total cholesterol(CHOL), were significantly elevated in the highest TyG index tertile(*p*<0.05 for all), reflecting broader metabolic abnormalities. Importantly, the prevalence of PTC increased significantly from T1 to T3 (54.8% vs.65.9%, p=0.007). These findings highlight the potential clinical utility of the TyG index as a biomarker for identifying individuals at increased risk of malignant thyroid nodules, particularly among Chinese adults with thyroid nodules.

**Table 1:** Population characteristics by categories of TyG index.

Characteristic	Total	T1(≤8.184)	T2(8.186-8.669)	T3(≥8.679)	*p*
No.	396	132	132	132	
Age, Mean(SD)	47.8 ± 12.7	44.3 ± 12.6	48.4 ± 12.8	50.8 ± 11.9	< 0.001
Sex, n (%)					< 0.001
Male	115 (29.0)	20 (15.2)	40 (30.3)	55 (41.7)	
Female	281 (71.0)	112 (84.8)	92 (69.7)	77 (58.3)	
Nodule aspect ratio, n (%)					0.651
<1	255 (64.4)	89 (67.4)	84 (63.6)	82 (62.1)	
≥1	141 (35.6)	43 (32.6)	48 (36.4)	50 (37.9)	
Nodule size(cm), n (%)					0.669
<1	145 (36.6)	52 (39.4)	48 (36.4)	45 (34.1)	
≥1	251 (63.4)	80 (60.6)	84 (63.6)	87 (65.9)	
Heart rate, Mean (SD)	78.1 ± 8.7	78.3 ± 9.8	77.3 ± 7.3	78.6 ± 8.8	0.399
Respiratory rate, Mean(SD)	19.6 ± 1.0	19.6 ± 1.0	19.6 ± 1.0	19.5 ± 0.9	0.834
BMI, kg/m^2^, Mean(SD)	24.6 ± 4.2	22.8 ± 3.3	24.6 ± 4.4	26.5 ± 4.1	< 0.001
SBP, mmHg, Mean(SD)	128.4 ± 17.4	122.3 ± 14.0	128.8 ± 18.4	134.2 ± 17.6	< 0.001
DBP, mmHg, Mean(SD)	80.9 ± 11.2	77.1 ± 10.5	81.2 ± 10.7	84.3 ± 11.1	< 0.001
Hypertension, n (%)					< 0.001
No	324 (81.8)	124 (93.9)	104 (78.8)	96 (72.7)	
Yes	72 (18.2)	8 (6.1)	28 (21.2)	36 (27.3)	
Other nodules, n (%)					0.938
No	342 (86.4)	115 (87.1)	113 (85.6)	114 (86.4)	
Yes	54 (13.6)	17 (12.9)	19 (14.4)	18 (13.6)	
iPTH, pg/mL, Median (IQR)	42.2 (33.7, 53.2)	40.3 (31.9, 53.2)	43.5 (34.0, 54.8)	43.3 (36.5, 50.9)	0.078
FT3, pmol/L, Mean(SD)	4.8 ± 0.7	4.7 ± 0.8	4.8 ± 0.6	4.8 ± 0.6	0.096
FT4, pmol/L, Mean(SD)	15.9 ± 2.5	15.8 ± 2.6	15.9 ± 2.4	16.1 ± 2.6	0.646
TSH, μIU/mL, Median (IQR)	1.9 (1.3, 2.9)	1.9 (1.3, 2.9)	1.9 (1.3, 2.8)	1.9 (1.3, 3.1)	0.851
CA, mmol/L, Mean(SD)	2.2 ± 0.1	2.2 ± 0.1	2.2 ± 0.1	2.3 ± 0.1	0.003
ALT, U/L, Median (IQR)	14.0 (10.0, 22.0)	11.0 (8.0, 14.0)	15.0 (11.0, 23.0)	19.0 (13.0, 29.0)	< 0.001
ALP, U/L, Mean(SD)	70.9 ± 22.5	63.7 ± 19.7	71.7 ± 22.1	77.3 ± 23.4	< 0.001
GGT, U/L, Median (IQR)	15.0 (11.0, 26.0)	11.0 (9.0, 15.0)	15.5 (11.0, 23.2)	26.0 (14.0, 38.2)	< 0.001
UA,μmol/L, Mean (SD)	287.5 ± 75.4	253.3 ± 62.3	288.7 ± 72.7	320.6 ± 75.5	< 0.001
CHOL, mmol/L, Mean (SD)	4.3 ± 0.8	4.1 ± 0.8	4.2 ± 0.8	4.5 ± 0.9	< 0.001
Outcome, n (%)					0.007
Benign	179 (45.2)	68 (51.5)	66 (50)	45 (34.1)	
Malignant	217 (54.8)	64 (48.5)	66 (50)	87 (65.9)	

Values were expressed as mean (standard deviation) or medians (T1-T3) or n (%).

SD, standard deviation; TyG, triglyceride glucose; BMI, body mass index; SBP, systolic blood pressure; DBP, diastolic blood pressure; iPTH, intact parathyroid hormone; FT3, free triiodothyronine; FT4, free thyroxine; TSH, thyroid-stimulating hormone; Ca, serum calcium; ALT, alanine aminotransferase; ALP, alkaline phosphatase; GGT, gamma-glutamyl transferase; UA, uric acid; CHOL, total cholesterol; OR, odds ratio; CI, conﬁdence interval; Ref, reference.

### Association between TyG index and papillary thyroid carcinoma

Univariate analysis showed that age, BMI, nodule aspect ratio, nodule size, TSH, ALT, TG, and TyG index were associated with PTC(*p*<0.1)(**Supplementary Table S1**).

**Figure 2:**
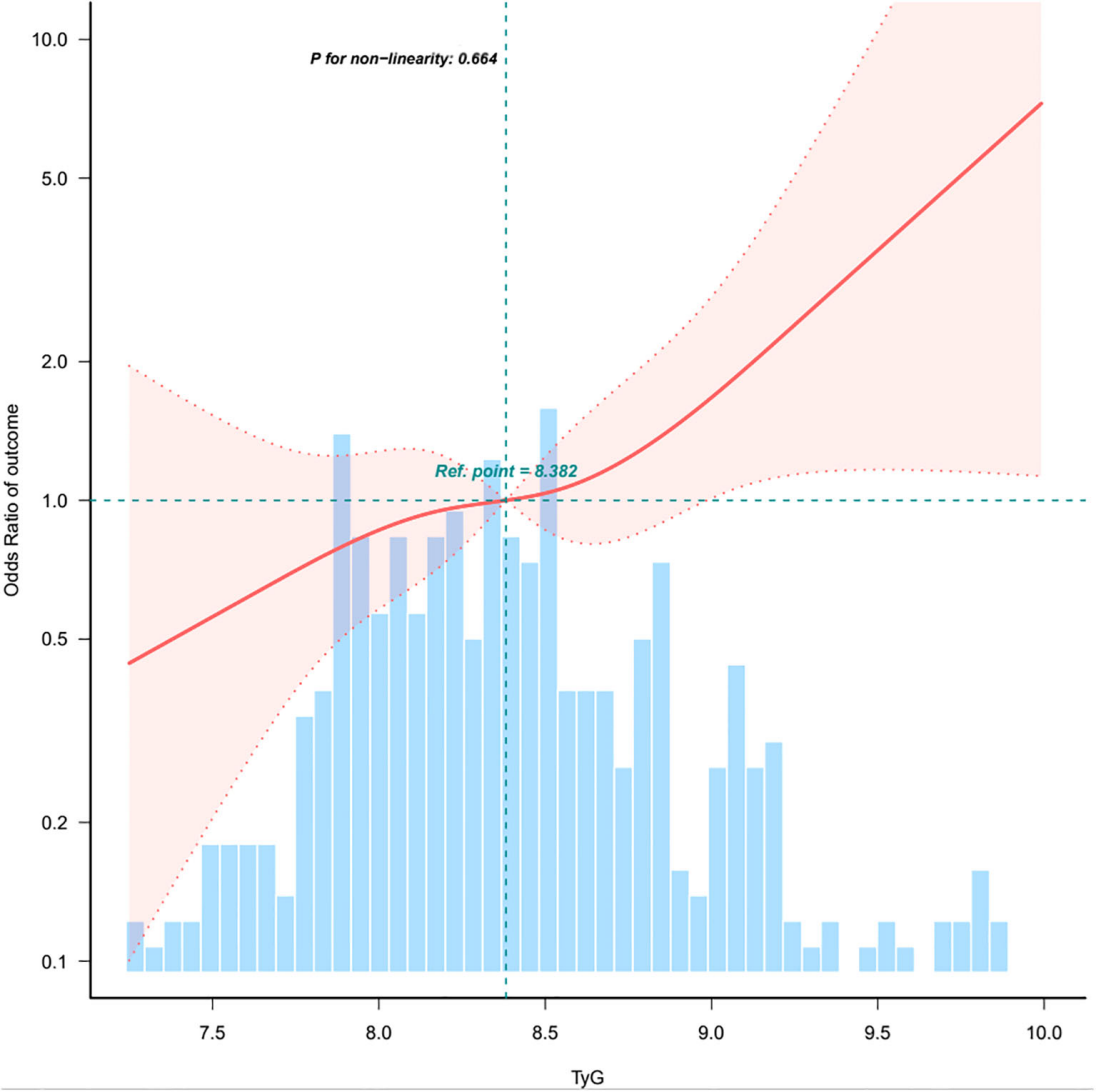
Association between TyG index and papillary thyroid carcinoma. CI, confidence interval; OR, odd ratio; RCS, restricted cubic spline. The model adjusted for age, sex, heart rate, respiratory rate, BMI, nodule aspect ratio, nodule size, hypertension, other nodules, Hashimoto’s thyroiditis, iPTH, intact parathyroid hormone; FT3, free triiodothyronine; FT4, free thyroxine; TSH, thyroid-stimulating hormone; ALT, alanine aminotransferase; ALP, alkaline phosphatase; UA, uric acid; CHOL, total cholesterol. Only 0.5-99.5% of the data is shown.

As shown in **Table 2**, each incremental point of the TyG index was associated with a 70% increase in the prevalence of PTC (OR = 1.70, 95%*CI*: 1.17-2.47, *p* = 0.005). This association remained significant in the fully adjusted model (OR = 2.25, 95%*CI*: 1.28-3.96, *p* = 0.005). When the TyG index was analyzed using tertiles, there was a significant positive association between the TyG index and PTC after adjusting for potential confounders. Compared with individuals with lower TyG index T1 (≤8.184), the adjusted OR values for TyG index and PTC in T2 (8.186-8.669), and T3 (≥8.679) were 1.28 (95%*CI*: 0.68-2.41, *p* = 0.443), 3.37(95%*CI*: 1.63-6.97, *p* = 0.001), respectively. To examine the linearity and further explore the shape of the dose-response relationship between the TyG index and PTC prevalence, restricted cubic spline analysis was conducted. Smooth curve fitting plots were generated based on the covariates in model 4. The analysis utilized four knots at the 5th, 35th, 65th, and 95th percentiles of the TyG index distribution (**Figure 2**). The estimated dose-response curve indicates a significant linear relationship between the TyG index and the risk of PTC (p for non-linearity=0.664).

**Table 2:** Association between the TyG index and papillary thyroid carcinoma.

TyG index tertiles	OR (95% CI)										
	No.	Crude	*p*	Model 1	*p*	Model 2	*p*	Model 3	*p*	Model 4	*p*
TyG index(Continuous variable)											
TyG index	217/396 (54.8%)	1.70 (1.17~2.47)	0.005	1.68 (1.08~2.62)	0.021	1.89 (1.15~3.11)	0.012	1.96 (1.19~3.24)	0.009	2.27 (1.29~3.97)	0.004
TyG index(Classified variable)											
T1(≤8.184)	64/132 (48.5%)	1(Ref)		1(Ref)		1(Ref)		1(Ref)		1(Ref)	
T2(8.186-8.669)	66/132 (50.0%)	1.06 (0.66~1.72)	0.806	1.11 (0.66~1.86)	0.691	1.12 (0.62~2.02)	0.696	1.17 (0.65~2.13)	0.598	1.28 (0.68~2.41)	0.443
T3(≥8.679)	87/132 (65.9%)	2.05 (1.25~3.37)	0.004	2.17 (1.23~3.84)	0.008	2.68 (1.39~5.15)	0.003	2.80 (1.44~5.44)	0.002	3.34 (1.62~6.90)	0.001
Trend.test			0.005		0.008		0.003		0.002		0.001

Crude: no adjusted; Model 1: age, sex, heart rate, respiratory rate, BMI; Model 2: Model 1+nodule aspect ratio and nodule size; Model 3: Model 2+hypertension, other nodules; Model 4: Model 3+iPTH, FT3, FT4, TSH, ALT, ALP, UA, CHOL.

TyG, triglyceride-glucose; BMI, body mass index; iPTH, intact parathyroid hormone; FT3, free triiodothyronine; FT4, free thyroxine; TSH, thyroid-stimulating hormone; ALT, alanine aminotransferase; ALP, alkaline phosphatase; UA, uric acid; CHOL, total cholesterol; OR, odds ratio; CI, confidence interval; Ref, reference.

### Subgroup analyses

**Figure 3:**
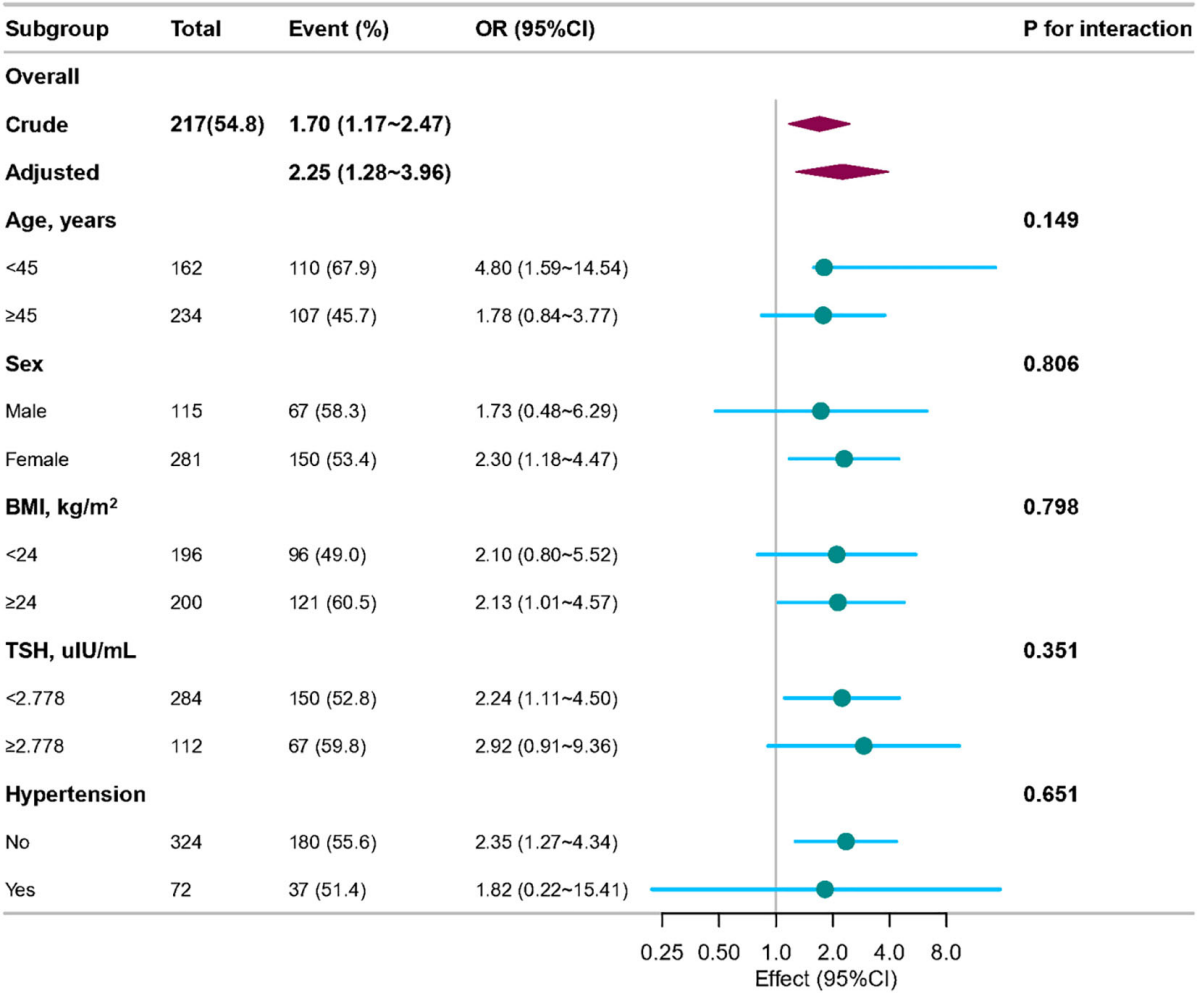
Association between TyG index and papillary thyroid carcinoma according to general characteristics. The stratifications were adjusted for all variables (age, sex, heart rate, respiratory rate, BMI, nodule aspect ratio and nodule size, hypertension, other nodules, Hashimoto’s thyroiditis, iPTH, FT3, FT4, TSH, ALT, ALP, UA, CHOL) except for the stratification factor itself. Circles represent the ORs and horizontal lines represent 95%CIs. CI, confidence interval; OR, odds ratio; TyG, triglyceride-glucose; BMI, body mass index; iPTH, intact parathyroid hormone; FT3, free triiodothyronine; FT4, free thyroxine; TSH, thyroid-stimulating hormone; ALT, alanine aminotransferase; ALP, alkaline phosphatase; UA, uric acid; CHOL, total cholesterol.

In order to detect whether the association between the TyG index and the risk of PTC exists in different subgroups, the analysis and interaction analysis were stratified according to confounding factors, including age, sex, BMI, thyroid-stimulating hormone (TSH), and hypertension (**Figure 3**). The subgroups showed no significant interaction (all p-values for interaction were greater than 0.05).

### Sensitivity analysis

After excluding all individuals with missing covariate values, 221 individuals remained, and the relationship between the TyG index and PTC remained stable. As a continuous variable, the TyG index was positively correlated with the incidence of PTC, with an odds ratio (OR) of 2.00(95%*CI*: 1.19-3.37, *p* = 0.009). After adjusting for potential confounding factors(model 4), the result remained consistent, with an OR of 3.51 (95%*CI*: 1.53-8.08, *p* = 0.003). When the TyG index was regarded as a categorical variable, compared with the individuals in the reference group(T1), the adjusted OR values for developing PTC were 1.75 (95% *CI*: 0.74-4.16, *p* = 0.206) in the T2 group(indicating a 75% increase in the risk of PTC) and 4.28 (95% *CI*: 1.59-11.52, *p* = 0.004) in the T3 group(indicating a 3.28-fold increase in the risk of PTC) (**Table 3**).

**Table 3:** Association between the TyG index and papillary thyroid carcinoma when individuals with missing data are all excluded.

TyG index tertiles	OR (95% CI)										
	No.	Crude	*p*	Model 1	*p*	Model 2	*p*	Model 3	*p*	Model 4	*p*
TyG index(Continuous variable)											
TyG index	133/221 (60.2%)	2.00 (1.19~3.37)	0.009	2.31 (1.24~4.29)	0.008	2.63 (1.31~5.28)	0.007	2.81 (1.38~5.75)	0.005	3.51 (1.53~8.08)	0.003
TyG index(Classified variable)											
T1(≤8.175)	39/74 (52.7%)	1(Ref)		1(Ref)		1(Ref)		1(Ref)		1(Ref)	
T2(8.184-8.598)	40/73 (54.8%)	1.09 (0.57~2.08)	0.799	1.48 (0.73~3.02)	0.277	1.56 (0.7~3.52)	0.279	1.62 (0.71~3.71)	0.25	1.75 (0.74~4.16)	0.206
T3(≥8.602)	54/74 (73.0%)	2.42 (1.22~4.81)	0.012	3.06 (1.4~6.69)	0.005	3.58 (1.48~8.63)	0.005	3.71 (1.53~9.04)	0.004	4.28 (1.59~11.52)	0.004
Trend.test			0.012		0.002		0.005		0.004		0.004

Crude: no adjusted; Model 1: age, sex, heart rate, respiratory rate, BMI; Model 2: Model 1+nodule aspect ratio and nodule size; Model 3: Model 2+hypertension, other nodules, Hashimoto’s thyroiditis; Model 4: Model 3+iPTH, FT3, FT4, TSH, ALT, ALP, UA, CHOL.

TyG, triglyceride-glucose; BMI, body mass index; iPTH, intact parathyroid hormone; FT3, free triiodothyronine; FT4, free thyroxine; TSH, thyroid-stimulating hormone; ALT, alanine aminotransferase; ALP, alkaline phosphatase; UA, uric acid; CHOL, total cholesterol; OR, odds ratio; CI, conﬁdence interval; Ref, reference.

## Discussion

This study found a significant link between a higher TyG index and increased PTC risk, which remained significant after adjusting for other confounding factors in multivariate regression analyses. Additionally, age, BMI, nodule aspect ratio and nodule size were closely related to PTC risk. These findings suggest that in clinical practice, both metabolic status and thyroid nodule characteristics should be considered for a more comprehensive PTC risk assessment. The TyG index reflects an individual’s state of insulin resistance and metabolic disturbance (32, 33). Insulin resistance can lead to elevated insulin levels, which in turn activate signaling pathways such as the insulin receptor substrate-1 (IRS-1) pathway, promoting thyroid cell proliferation and carcinogenesis (34). In a metabolically disordered state, increased oxidative stress generates more free radicals, which can damage thyroid cell DNA and trigger gene mutations, thereby raising the risk of PTC (35). Reactive oxygen species (ROS) can attack DNA, causing base damage and DNA strand breaks. If these injuries are not promptly repaired, they may lead to cell cycle dysregulation and oncogene activation, fostering thyroid cancer development (36). Accumulation of inflammatory cells and cytokines in the tumor microenvironment can promote the proliferation and invasion of thyroid cancer cells (37). Chronic inflammation can also alter the thyroid tissue microenvironment, creating favorable conditions for cancer cell expansion and metastasis (37, 38).

Elevated triglyceride levels can supply energy and biosynthetic precursors for cancer cells (39). With their high metabolic demands, tumor cells can use fatty acids from triglyceride breakdown as energy sources and for maintaining cell membrane homeostasis (40, 41). These lipid abnormalities can fuel the rapid proliferation and metabolic reprogramming of cancer cells, thus sustaining PTC progression (42). Additionally, hyperglycemia may affect thyroid hormone-binding protein levels, affecting thyroid hormone transport and efficacy (43).

This study found a significant association between an elevated TyG index and increased PTC risk, a result that aligns with the findings of Alkurt et al., who reported that the TyG index was significantly higher in the malignant thyroid-disease group than in the benign group. ROC-curve analysis showed that the TyG index has predictive value for distinguishing papillary thyroid carcinoma from non-malignant thyroid lesions (AUC: 0.608). At a cut-off of 6.252, sensitivity and specificity were 62.8% and 49.2%, respectively (25). These data suggest that the TyG index may serve as a useful tool for identifying individuals at high risk of papillary thyroid carcinoma. Wang and colleagues’ Mendelian randomization study further supports a causal relationship between metabolic syndrome and thyroid cancer, demonstrating that genetically predicted metabolic syndrome is positively associated with increased thyroid-cancer risk (44). Nevertheless, conflicting results regarding the TyG–PTC link have been reported. Kim et al. observed that while METS-IR (metabolic score for insulin resistance) was positively associated with thyroid-cancer incidence, this relationship was more pronounced in the subgroup with BMI < 25 kg/m² (45). These findings imply that obesity status may modulate the relationship between insulin resistance and thyroid cancer.

However, this study has several limitations. First, this research is based on a single-center cross-sectional study only captures a specific time point, so it cannot establish causality. In the future, we will carry out a multicenter study in Jiangsu, increasing the sample size and collecting more related factors like TI-RADS scores, echogenicity, cardiovascular disease and metabolic syndrome to conduct a more comprehensive study. Secondly, like other studies, it’s difficult to rule out unmeasured variables or unknown confounding factors. Also, this study doesn’t include metabolic syndrome, which involves various metabolic abnormalities that may affect thyroid cancer risk. This means the study results might not fully capture the relationship between metabolic factors and PTC development. Future research should include metabolic syndrome to address this limitation. Lastly, measurement errors can occur in cross-sectional studies. For example, TyG index calculation relies on tests of fasting blood glucose and triglycerides, which can be influenced by testing methods and diet, potentially affecting result accuracy. Patients fasted for over 12 hours before blood collection to reduce bias. Also, various batches of TG and GLU reagents influenced the test results somewhat. To ensure reliability, internal quality control (IQC) is implemented to check results before testing clinical samples. Our laboratory has participated in the NCCL’s External Quality Assessment (EQA) program three times annually since 2007, and all EQA results were satisfactory during this study. Moreover, the Roche c701 biochemistry analyzer must be calibrated twice a year as part of regular maintenance. Therefore, all the testing results were reliable. In addition, our laboratory has also obtained ISO(International Organization for Standardization) 15189 medical laboratory accreditation from the China National Accreditation Service for Conformity Assessment (CNAS). The accredited items include TG and GLU.

## Conclusion

In conclusion, our study has revealed a significant association between the TyG index and PTC risk in Chinese adults with thyroid nodules. In the study population, calculating the TyG index using routinely available laboratory data may help identify high-risk individuals for PTC among Chinese patients with thyroid nodules. This study suggests that TyG index may serve as a valid biomarker for predicting PTC in patients with thyroid nodules, although large prospective studies are needed to confirm these findings.

## Data Availability

The data analyzed in this study is subject to the following licenses/restrictions: The raw data needed to recreate these results can’t be shared right now since it’s part of an ongoing study. However, if needed, some or all of the data from this study can be obtained by contacting the corresponding author. Requests to access these datasets should be directed to tonghc0716@126.com.
