# Supplementary material for: Association between triglyceride-glucose index and papillary thyroid carcinoma among Chinese adults with thyroid nodules
Source: Front Endocrinol (Lausanne). 2025 Sep 25;16:1616350. doi: 10.3389/fendo.2025.1616350 (PMC12507552; doi:10.3389/fendo.2025.1616350)
Supplement: Supplementary file 1 [file Table1.docx]

**Supplementary Materials Files**

**To: **Association between Triglyceride-Glucose Index (TyG) and papillary thyroid carcinoma among Chinese adults with thyroid nodules****

**by Chunyan Song, Xing Meng, Yun Lan, Miaomiao Ping, Ling Lin, Huacheng Tong**

**Supplementary Table**

**Supplementary Table 1. Associations between variables and papillary thyroid carcinoma.**

| Variable | OR(95*CI%*) | *P* |
| --- | --- | --- |
| Age | 0.97 (0.96,0.99) | 0.001 |
| Sex:Female vs.male | 0.78 (0.51,1.19) | 0.247 |
| BMI(kg/m^2^) | 1.09 (1.03~1.14) | 0.001 |
| Nodule aspect ratio, n (%)  (≥1 vs.＜1） | 8.49 (5.16,13.96) | <0.001 |
| Nodule size(cm), n (%),  ( ≥1 vs.＜1） | 0.28 (0.18,0.43) | <0.001 |
| Heart rate（minute） | 1.01 (0.99,1.03) | 0.516 |
| Respiratory rate (minute） | 1.04 (0.86,1.27) | 0.674 |
| SBP(mmHg) | 1.00 (0.99,1.01) | 0.679 |
| DBP(mmHg) | 1.01 (1.00,1.03) | 0.138 |
| Hypertension,yes vs. no | 1.09 (0.69,1.74) | 0.708 |
| Qther nodules,yes vs. no | 1.07 (0.62,1.85) | 0.801 |
| Hashimoto's thyroiditis,yes vs. no | 0.41 (0.16,1.04) | 0.061 |
| iPTH (pg/mL) | 1.00 (1.00,1.01) | 0.541 |
| FT3 (pmol/L) | 0.90 (0.68,1.18) | 0.443 |
| FT4 (pmol/L) | 0.99 (0.92,1.07) | 0.881 |
| TSH (μIU/mL) | 1.18 (1.04,1.35) | 0.012 |
| CA (mmol/L) | 0.62 (0.10,3.82) | 0.608 |
| ALT (U/L) | 1.02 (1.00,1.03) | 0.015 |
| ALP (U/L) | 1.00 (0.99,1.01) | 0.93 |
| GGT (U/L) | 1.00 (1.00,1.01) | 0.28 |
| UA (μmol/L) | 1.00 (1.00,1.00) | 0.382 |
| CHOL (mmol/L) | 0.91 (0.72,1.14) | 0.398 |
| TG (mmol/L) | 1.55 (1.19,2.02) | 0.001 |
| GLU (mmol/L) | 1.18 (0.96,1.44) | 0.108 |
| TyG Index | 1.70 (1.17,2.47) | 0.005 |

Abbreviations: BMI,body mass index,iPTH, intact parathyroid hormone;FT3,free triiodothyronine; FT4,free thyroxine;TSH,thyroid-stimulating hormone;CA,serum calcium;ALT,alanine aminotransferaseserum;ALP,alkaline phosphatase calcium;GGT,gamma-glutamy;UA,uric acid; CHOL,total cholesterol;TG,triglycerides;GLU,blood glucose;TyG,triglyceride-glucose index;OR, odds ratio;CI,conﬁdence interval;
